# Spread of *Neisseria meningitidis* Serogroup W Clone, China

**DOI:** 10.3201/eid1909.130160

**Published:** 2013-09

**Authors:** Haijian Zhou, Wei Liu, Li Xu, Lili Deng, Qiuyun Deng, Jiatong Zhuo, Zhujun Shao

**Affiliations:** National Institute for Communicable Disease Control and Prevention, Beijing, People’s Republic of China (H. Zhou, L. Xu, Z. Shao);; State Key Laboratory for Infectious Disease Prevention and Control, Beijing (H. Zhou, Z. Shao);; Guangxi Center for Disease Control and Prevention, Nanning, People’s Republic of China (W. Liu, L. Deng, Q. Deng, J. Zhuo)

**Keywords:** Neisseria meningitidis, serogroup W135, meningococcal infections, multilocus sequence typing, China, bacteria

## Abstract

During February 2011–June 2012, invasive infection with *Neisseria meningitidis* serogroup W was identified in 11 persons in southeastern China. All isolates tested had matching or near-matching pulsed-field gel electrophoresis patterns and belonged to multilocus sequence type 11. The epidemiologic investigation suggested recent transmission of this clonal complex in southeastern China.

*Neisseria meningitidis* is a major public health threat in many parts of the world, including China. Since 2003, most meningococcal diseases in China have been caused by *N. meningitidis* serogroups A and C; only 3 cases of serogroup W meningococcal disease were reported before 2011 (*1*,*2*). However, during February 2011–June 2012, an increase in invasive disease caused by serogroup W *N. meningitidis* (11 cases total) was seen in southeastern China. To determine if this serogroup is emerging in China, we analyzed strains from 6 of the 11 infected patients reported during 2011–2012, from 16 of their close contacts, and from 3 serogroup W patients reported during 2006–2008.

## The Study

Meningococcal disease is reportable in China. *N. meningitidis* isolates, cerebrospinal fluid (CSF), and blood samples from persons with invasive disease are forwarded to the Chinese Centers for Disease Control and Prevention (CDC) for serogroup determination by slide agglutination and/or PCR. Strains are further characterized by use of pulsed-field gel electrophoresis (PFGE) after *Nhe*I restriction enzyme digestion (*3*).

During February 2011–June 2012, we observed an increase in invasive meningococcal disease caused by *N. meningitidis* serogroup W in southeastern China. Of 11 cases total, 9 were diagnosed by strain isolation and 2 by PCR and real-time PCR of CSF samples (Table). Strains isolated from patients 1, 4, and 5 became nonviable during storage in the hospital laboratory. The 6 remaining strains (from patients 2, 3, 7, and 9–11) were submitted to the Chinese CDC along with 16 serotype W strains from close contacts of patients 4, 6, and 8–10. Thus, during 2011–2012, a total of 22 strains were submitted to the Chinese CDC, where they were confirmed as *N. meningitidis* serogroup W by slide agglutination with specific antiserum (Remel, Lenexa, KS, USA). In addition, CSF samples from patients 1, 4–6, and 8 were submitted and confirmed positive for *N. meningitidis* serogroup W by PCR and real-time PCR.

**Table:** Clinical and epidemiologic characteristics of patients with ST11 serogroup W meningococcal disease, China, 2011–2012*

Patient ID	Age, y/sex	Date of symptom onset	Province of onset	Patient occupation	Outcome	Method of diagnosis	ST11 W strains from close contacts (no.)
1†	16/M	2011 Feb 12	Guangxi	Student	Survived	Strain isolation/ PCR	No
2	19/M	2011 Apr 1	Jiangsu	Factory worker	Died	Strain isolation	No
3	19/F	2011 Apr 20	Zhejiang	Factory worker	Died	Strain isolation	No
4†	18/M	2011 Apr 27	Guangxi	Student	Survived	Strain isolation/ PCR	Yes (8)
5†	46/M	2011 May 4	Guangxi	Farmer	Survived	Strain isolation/ PCR	No
6†	22/F	2011 Oct 13	Guangdong	Factory worker	Survived	PCR	Yes (1)
7	35/M	2012 Feb 1	Guangdong	Factory worker	Survived	Strain isolation	No
8†	23/M	2012 Feb 2	Guangxi	Factory worker	Survived	PCR	Yes (1)
9	14/M	2012 Feb 14	Anhui	Student	Died	Strain isolation	Yes (4)
10	3/M	2012 Mar 27	Henan	Student	Survived	Strain isolation	Yes (2)
11	9/M	2012 Jun 18	Hunan	Student	Survived	Strain isolation	No

*ID, identification; ST, sequence type. †Multilocus sequence typing results were obtained from cerebrospinal fluid but not from strains.

**Figure 1:**
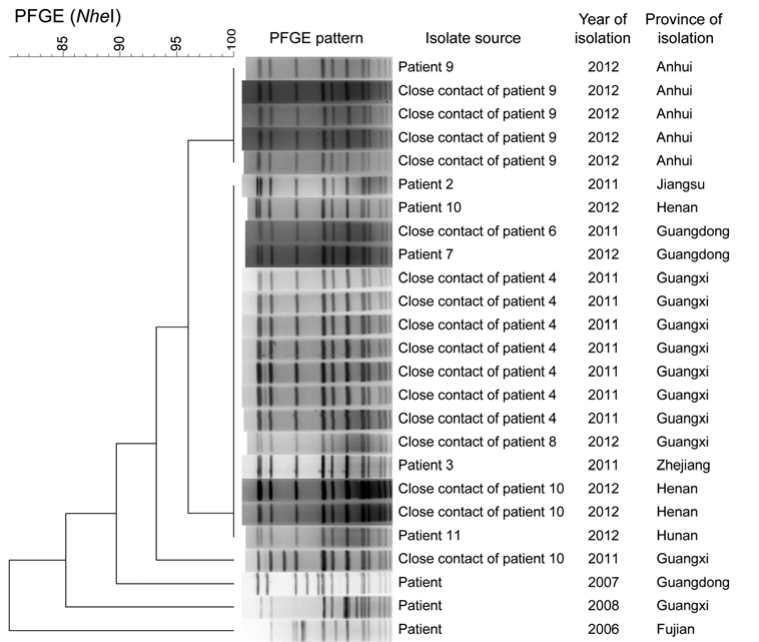
PFGE patterns for 22 *Neisseria meningitidis* serogroup W strains (6 from reported patients, 16 from close contacts) isolated during 2011–2012 and 3 isolated during 2006–2008, China. All isolates were sequence type 11 (determined by multilocus sequence typing) and PorA type P1.5,2. PFGE, pulsed-field gel electrophoresis. Scale bars indicates percent similarity.

The 22 serogroup W strains from 2011–2012 were analyzed by PFGE; for comparison, 3 strains isolated from patients during 2006–2008 were also analyzed. PFGE patterns were distinguishable for 16 of the 22 strains from 2011–2012. Five strains associated with patient 9 and 1 strain isolated from a close contact of patient 4 had PFGE patterns that differed by 1 and 2 bands, respectively, indicating >94% similarity with the dominant pattern (Figure 1). PFGE patterns for the 3 isolates from 2006–2008 exhibited <90% similarity with those for isolates from 2011–2012, differing by 4–7 bands. All 22 isolates and 5 CSF samples from 2011–2012 were identified as sequence type (ST) 11 and PorA type P1.5,2, identical to the genotype of serotype W isolates associated with outbreaks reported in Saudi Arabia in 2000 and 2001 (*4*,*5*) and Burkina Faso in 2002 (*6*), sporadic cases in other countries (*7*–*9*), and the 3 cases identified in China during 2006–2008 (*2*).

Of the 11 patients with cases reported during 2011–2012, 4 resided in Guangxi Province, 2 in Guangdong Province, and 1 each in Jiangsu, Zhejiang, Anhui, Henan, and Hunan Provinces (Technical Appendix). The median age of patients was 20 years (range 3–46), 9 (81.8%) were male, and all denied recent foreign travel. Three of the 11 patients died of bacteremia. The epidemiologic investigation did not identify any common exposures, social settings, or other connections among the patients. Close contacts of all 11 patients were investigated, and no additional *N. meningitidis* infections were detected. Of the 11 reported cases, 5 occurred in or were associated with Laibin City, Guangxi Province: patients 1, 4, and 5 sought care in Laibin City; patient 3 sought care in Zhejiang Province on April 20, 2012, after having traveled to Zhejiang Province from Laibin City on March 26; and patient 8 sought care in Fangchenggang City, Guangxi Province, 10 days after a close contact (partner) had traveled to Laibin City (Technical Appendix). The partner of patient 8 was subsequently tested and identified as a carrier of ST11 serogroup W *N. meningitidis*.

A survey of *N. meningitidis* carriage was conducted among the healthy population of Laibin City in September 2011. A total of 1,311 persons 1–45 years of age were investigated, of whom 8.54% (112/1,311 persons) were positive for *N. meningitidis* carriage. Age groups and percentages of infected persons in each age group were 1–6 years (1.4%), 7–12 years (6.0%), 13–15 years (6.7%), 16–20 years (18.5%), and 21–45 years (1.0%). The serogroup for each strain was determined by use of slide agglutination and polyclonal antisera and PCR methods. Of the 112 *N. meningitidis*–positive samples, 20 (17.9%) were ST11 serogroup W, and of those 20 samples, 2, 4, and 14 were from persons 7–12, 13–15, and 16–20 years of age, respectively. All 20 strains exhibited indistinguishable PFGE patterns that matched the dominant pattern of the disease-associated strains. The carriage rate of ST11 serogroup W *N. meningitidis* reached 5.5% (11/200) among 200 students (16–20 years of age) in 1 school.

**Figure 2:**
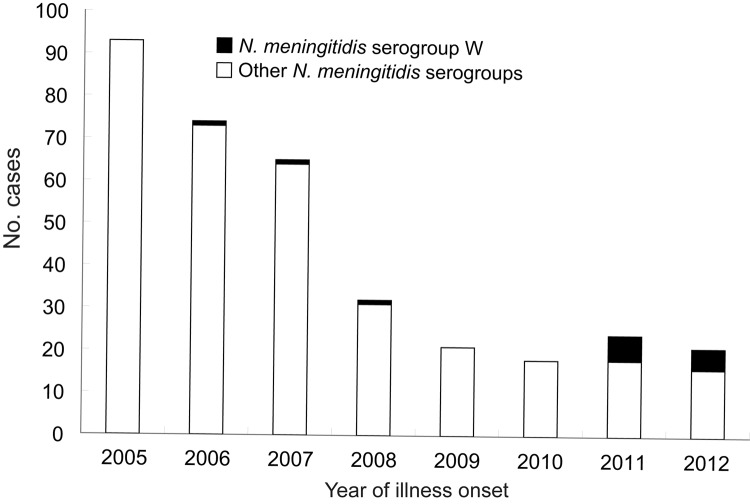
Laboratory-confirmed cases of meningococcal disease, by *Neisseria meningitidis* serogroup and year of symptom onset, China, 2005–2012.

Since 2003, the annual incidence of meningococcal disease in China has stayed below 0.2 cases/100,000 population. Surveillance data in China suggest a historical trend for seasonal peaks of meningococcal disease during February–April. This peak season corresponds with the spring dry season in China, a time when tourists are most likely to visit the country, especially southern China. Among the 45 cases of meningococcal disease confirmed during 2011–2012 by meningococcal etiology and PCR methods, 11 (24.4%) were caused by serogroup W *N. meningitidis* (Figure 2); 8 of these 11 cases occurred during February–April. The 3 cases reported during 2006–2008 occurred during May, June, and October, respectively.

## Conclusions

The incidence of serogroup W infections reported during February 2011–June 2012 represents a marked increase over that reported during 2005–2010. The emergence and spread of a new *N. meningitidis* serogroup in a region presents a challenge for the prevention and control of meningococcal disease, especially if vaccines used in the region do not cover all serogroups. ST7 serogroup A and ST4821 serogroup C *N. meningitidis* strains were identified as the 2 dominant lineages circulating in China during 2003–2008, causing >90% of meningococcal disease cases (*1*). Meningococcal polysaccharide vaccines A and C have been used in China for routine immunization since the outbreak of *N*. *meningitidis* serogroup C during 2003–2004. In some African countries, repeated vaccination against *N. meningitidis* serogroups A and C is thought to have led to a selective increase in the incidence of meningococci of other serogroups, thereby resulting in a changed profile of meningococcal disease (*10*–*12*). Therefore, meningococcal disease caused by *N. meningitidis* strains that belong to serogroups other than A and C, especially those that belong to hyperinvasive lineages, appears to be an emerging problem in China. 

The 11 cases of meningococcal disease caused by ST11 serogroup W *N. meningitidis* strains described here had successively emerged in southeastern China; furthermore, ST11 serogroup W meningococci were isolated from close contacts of the patients and from healthy carriers. These observations suggest the possible establishment and spread of a clonal complex of serogroup W meningococci in southeastern China. Carriage and transmission of this strain have led to the emergence of ST11 serogroup W organisms as a cause of endemic meningococcal disease. Further epidemiologic and microbiological surveillance is needed for monitoring of meningococcal diseases caused by serogroup W in southeastern China and preventing the spread of this clone to other regions.

Technical Appendix Figure showing distribution of 11 serogroup W meningococcal disease cases identified in China during February 2011–June 2012.

## References

[1] Zhou H, Gao Y, Xu L, Li M, Li Q, Li Y, Distribution of serogroups and sequence types in disease-associated and carrier strains of *Neisseria meningitidis* isolated in China between 2003 and 2008. Epidemiol Infect. 2012;140:1296–303 . 10.1017/S0950268811001865 21929839

[2] Shao Z, Zhou H, Gao Y, Ren H, Xu L, Kan B, *Neisseria meningitidis* serogroup W135, China. Emerg Infect Dis. 2010;16:348–9 . 10.3201/eid1602.090901 20113581 PMC2958009

[3] Shao Z, Li W, Ren J, Liang XF, Xu L, Diao BW, Identification of a new *Neisseria meningitidis* serogroup C clone from Anhui Province, China. Lancet. 2006;367:419–23. 10.1016/S0140-6736(06)68141-5 16458767

[4] Lingappa JR, Al-Rabeah AM, Hajjeh R, Mustafa T, Fatani A, Al-Bassam T, Serogroup W-135 meningococcal disease during the Hajj, 2000. Emerg Infect Dis. 2003;9:665–71. 10.3201/eid0906.020565 12781005 PMC3000138

[5] Mayer LW, Reeves MW, Al-Hamdan N, Sacchi CT, Taha MK, Ajello GW, Outbreak of W135 meningococcal disease in 2000: not emergence of a new W135 strain but clonal expansion within the electrophoretic type-37 complex. J Infect Dis. 2002;185:1596–605. 10.1086/340414 12023765

[6] Nathan N, Rose AM, Legros D, Tiendrebeogo SR, Bachy C, Bjørløw E, Meningitis serogroup W135 outbreak, Burkina Faso, 2002. Emerg Infect Dis. 2007;13:920–3. 10.3201/eid1306.060940 17553237 PMC2792856

[7] Lemos AP, Harrison LH, Lenser M, Sacchi CT. Phenotypic and molecular characterization of invasive serogroup W135 *Neisseria meningitidis* strains from 1990 to 2005 in Brazil. J Infect. 2010;60:209–17. 10.1016/j.jinf.2009.11.014 20056121 PMC4631377

[8] Doyle TJ, Mejia-Echeverry A, Fiorella P, Leguen F, Livengood J, Kay R, Cluster of serogroup W135 meningococci, southeastern Florida, 2008–2009. Emerg Infect Dis. 2010;16:113–5. 10.3201/eid1601.091026 20031054 PMC2874373

[9] Kilic A, Urwin R, Li H, Saracli MA, Stratton CW, Tang YW. Clonal spread of serogroup W135 meningococcal disease in Turkey. J Clin Microbiol. 2006;44:222–4. 10.1128/JCM.44.1.222-224.2006 16390974 PMC1351935

[10] Fonkoua MC, Taha MK, Nicolas P, Cunin P, Alonso JM, Bercion R, Recent increase in meningitis caused by *Neisseria meningitidis* serogroups A and W135, Yaoundé, Cameroon. Emerg Infect Dis. 2002;8:327–9. 10.3201/eid0803.010308 11927034 PMC2732466

[11] Gagneux SP, Hodgson A, Smith TA, Wirth T, Ehrhard I, Morelli G, Prospective study of serogroup X *Neisseria meningitidis* outbreak in northern Ghana. J Infect Dis. 2002;185:618–26 . 10.1086/339010 11865418

[12] Massenet D, Inrombe J, Mevoula DE, Nicolas P. Serogroup W135 meningococcal meningitis, northern Cameroon, 2007–2008. Emerg Infect Dis. 2009;15:340–2. 10.3201/eid1502.080988 19193290 PMC2662656

