# Supplementary material for: Spread of Neisseria meningitidis Serogroup W Clone, China
Source: Emerg Infect Dis. 2013 Sep;19(9):1496–9. doi: 10.3201/eid1909.130160 (PMC3810921; doi:10.3201/eid1909.130160)
Supplement: Technical Appendix — Figure showing distribution of 11 serogroup W meningococcal disease cases identified in China during February 2011–June 2012. [file 13-0160-Techapp-s1.pdf]

Article DOI: http://dx.doi.org/10.3201/eid1909.130160

Clonal Spread of Serogroup W Meningococcal Disease in China

Technical Appendix

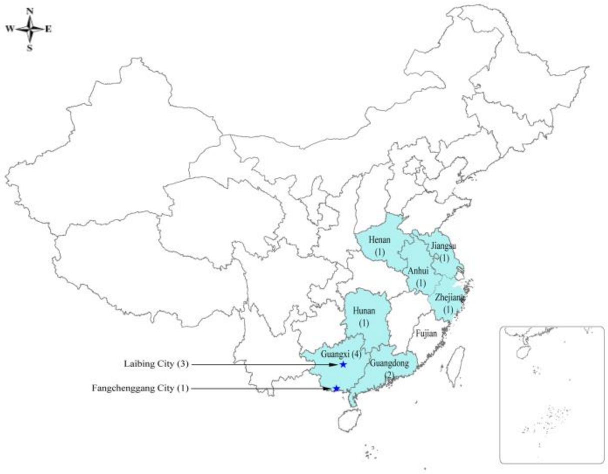

Technical Appendix Figure. Distribution of 11 serogroup W meningococcal disease cases identified in China during February 2011–June 2012. Numbers in parentheses indicate the number of confirmed cases from each region.
